# Ionic Twin Nanostructural Comparison: Propylammonium Butanoate vs. Butylammonium Propanoate and Their Interactions with Water

**DOI:** 10.3390/ma17164071

**Published:** 2024-08-16

**Authors:** Umme Salma, Natalia V. Plechkova, Lorenzo Gontrani, Marilena Carbone

**Affiliations:** 1 CNR NANOTEC—Institute of Nanotechnology, Via per Monteroni, 73100 Lecce, Italy; 2 Wellcome-Wolfson Institute for Experimental Medicine, The Queen’s University of Belfast, 97 Lisburn Road, Belfast BT9 7BL, UK; 3 Department of Chemical Science and Technologies, University of Rome Tor Vergata, Via della Ricerca Scientifica 1, 00133 Roma, Italy; lorenzo.gontrani@uniroma2.it (L.G.); carbone@uniroma2.it (M.C.)

**Keywords:** ionic liquids, MD simulations, X-ray scattering, nanostructure, PILs

## Abstract

This study investigates the nanostructure of two protic ionic liquids (PILs), [N_0 0 0 3_][C_3_CO_2_] and [N_0 0 0 4_][C_2_CO_2_], with similar polar head groups but varying alkyl chain lengths. An X-ray scattering technique and molecular dynamics simulations have been utilized to characterize the bulk and interfacial properties of these PILs. The findings suggest that the nanostructure of the PILs is primarily determined by the electrostatic forces between charged functional groups playing a dominant role. Despite differences in the alkyl chain lengths, the PILs possess remarkably similar nanostructures. Extending our investigation, we report the impact of water on the nanostructure. Our findings reveal that the addition of water disrupts interactions between cations and anions, weakening Coulombic forces. The disruptive behavior is attributed to the establishment of hydrogen bonds between water and ions. This comprehensive approach provides valuable insights into the nuanced factors shaping the nanostructure of these PILs, which are crucial for tailoring their applications in synthetic chemistry, catalysis, and beyond.

## 1. Introduction

**Figure 1:**
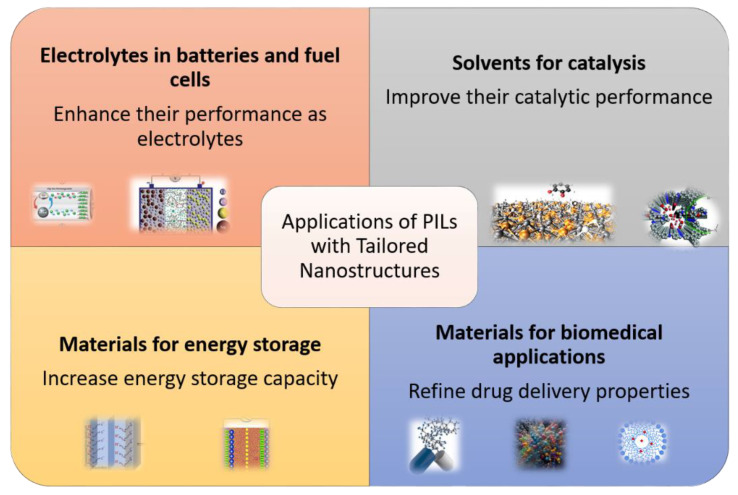
Some potential applications of PILs with tailored nanostructures.

Protic ionic liquids (PILs) are a class of ionic liquids (ILs) that are formed by the proton transfer between Brønsted acids and bases [1]. Due to their special qualities and prospective uses in a variety of industries, including catalysis, electrochemistry, biotechnology, and nanotechnology, PILs have garnered a lot of interest [2,3]. PILs exhibit a complex nanostructure that arises from the interplay of Coulombic and van der Waals interactions between their constituent ions [4]. Investigating the nanostructure of these systems plays a pivotal role in determining the behavior and enhanced performance of ionic liquids, especially in applications that rely on their unique properties [1,2,5]. Nanostructure impacts key physical and chemical aspects such as viscosity, ionic conductivity, thermal stability, and solvation of catalysts. The ability to precisely manipulate the nanostructure of PILs can lead to, i.e., more efficient energy storage systems, faster catalytic reactions, and targeted drug delivery with reduced side effects [6,7]. Applications of PILs possessing nanostructures are shown in Figure 1. Recently, a comprehensive review on the applications and prospects of ILs towards the chemistry of biomolecules, covering the latest advances and challenges in this field, was published [8]. Similarly, small-angle neutron scattering (SANS) and molecular dynamics simulations were used to study the micellar structures and aggregation numbers of sodium dodecyl sulfate (SDS) in different solvents, such as water, ionic liquids, and water–ionic liquid mixtures. Researchers found that the nanostructure of the solvent can modulate the hydrophobic and electrostatic interactions between the surfactant molecules and affect the micellar size and shape in ionic liquids [9]. Molecular dynamics simulations and X-ray scattering experiments were used to show that mixing an ionic liquid or ionic liquid electrolyte with a non-solvating fluorous diluent produces a low-viscosity mixture in which the local ion arrangements, and therefore key physical properties, are retained or enhanced [10]. It was also shown that the nanostructure of the locally concentrated ionic liquids and ionic liquid electrolytes is largely preserved in bulk and at gold and graphite electrodes for all potentials investigated [10].

The molecular structure of PILs is a crucial factor that influences their nanostructure [11]. The polar head groups and alkyl chain lengths in PILs play a significant role in determining the size, shape, packing, and ordering of the polar and non-polar domains in PILs. Previous studies have shown that the polar head groups determine the size and shape of the polar domains, while the alkyl chain lengths and positions on ions affect the packing and ordering of the non-polar domains [12,13]. However, most of these studies have focused on comparing PILs with different polar head groups or different alkyl chain lengths of cation and anion, while few have explored the effect of changing both factors simultaneously [4].

One review discusses how small changes in the chemical structure of ionic liquids, including variations in alkyl chain length, can influence their nanostructure formation and self-assembly behavior. This review highlights that even minor changes can lead to significant differences in physical properties such as viscosity, conductivity, and thermal stability [6]. Another study by Canongia Lopes and Padua investigates how varying the alkyl chain length of ionic liquids affects their nanostructures. They found that even a single carbon difference can alter the self-assembly behavior, though these changes might not always be dramatic. This indicates that small differences in alkyl chain length can still impact nanostructural arrangements [14]. Molecular dynamics simulations were used to explore how different alkyl chain lengths impact the structure and dynamics of ionic liquids. The findings suggested that while longer alkyl chains have more pronounced effects, even shorter chains can show noticeable differences in nanostructure formation, supporting the idea that even small variations matter [15]. In a comparative study, it was reported that ionic liquids with alkyl chains differing by just one carbon atom can exhibit comparable but subtly different nanostructures [16]. These differences in molecular organization and packing can influence macroscopic properties, suggesting that detailed analysis is crucial to fully understand the impact of such small variations.

Despite the wealth of research in this area, there is a gap in understanding how variations in alkyl chain lengths influence the overall organization of PILs, especially when the polar head groups are kept the same. Addressing this gap is crucial for tailoring PILs with specific properties for targeted applications. To bridge this knowledge gap, this study investigates how the molecular structure of two PILs affects their nanostructure in both bulk and interfacial environments. The PILs investigated are propylammonium butanoate ([N_0 0 0 3_][C_3_CO_2_]) and butylammonium propanoate ([N_0 0 0 4_][C_2_CO_2_]), which have the same polar alkylammonium head groups but different alkyl chain lengths and positions of the cations and anions. The total number of carbon atoms in the alkyl chains of cation and anion is four or three for both PILs, but in reverse order: [N_0 0 0 3_][C_3_CO_2_] has the propylammonium cation and the butanoate anion, while [N_0 0 0 4_][C_2_CO_2_] has the butylammonium cation and the propanoate anion. This paper uses experimental and computational methods to show that the polar head groups have a more significant influence on the nanostructure of the PILs than the alkyl chain lengths or their position. This study also shows that the chain lengths in cations and anions of the PILs does not affect the X-ray scattering patterns, as the peaks associated with the non-polar and polar domains of the PILs overlap. 

## 2. Materials and Methods

### 2.1. Synthesis

To an equimolar volume of an aqueous alkylamine solution, propanoic and butanoic acids (Sigma-Aldrich, St. Louis, MO, USA, ≥99.5% and ≥99% purities, respectively) were added dropwise. Sigma-Aldrich provided the butylamine (99.5% purity) and propylamine (99.0% purity). Liquid nitrogen (N_2_) was used to chill the one-neck 1-1 round-bottom flasks in which the reactions were conducted. The flasks were sealed and maintained in a liquid N_2_ atmosphere following each injection of acid, and extra cooling was achieved by adding more liquid N_2_ as needed. Since the reactions are exothermic, it was essential to keep the temperature low. The reactions were allowed to come to room temperature for about two hours after all the acids were added, and they were then agitated for an hour at room temperature.

The subsequent steps of the synthesis were as follows:

- Karl Fischer titration was used to determine the water content following a 12 h cycle.
- Water was extracted using a freeze-drying process at 0.03 mbar pressure. The freeze-drying cycle was carried out twice more, if the water mole percentage was greater than 0.1, or until no decrease in the water content was noticed.

In the glove box, in a dry N_2_ environment, the resulting bright yellow, highly hygroscopic [N_0 0 0 3_][C_3_CO_2_] (97% yield) and [N_0 0 0 4_][C_2_CO_2_] (98% yield) salts were stored until needed. Over a two-year period, despite color changes, the X-ray diffraction patterns revealed that the ionic liquids’ structures remained substantially unaltered. This implies that the extremely small number of breakdown products had no bearing on the liquids’ structural behavior.

### 2.2. Methods

#### 2.2.1. X-ray Scattering

A Bruker AXS D8 Advance powder diffractometer (Billerica, MA, USA) in transmission mode with ϑ/ϑ geometry was used for small-angle diffraction investigations. Samples were put into SiO_2_-glass capillaries with a 2 mm diameter, and Parafilm was used to close the charging funnels. These capillaries were positioned along the beam path and firmly attached, inverted, onto a conventional goniometer head using beeswax. The apparatus is furnished with a VÅntec-1 position-sensitive detector (PSD), focusing Göbel mirrors along the incident beam, and Soller slits on both incident (2.3° horizontal divergence) and diffracted (radial) beams. In step-scan mode, data were obtained in the 2–40° 2 ϑ angular range, with a counting time of one second and a step size of 0.022° 2 ϑ. The source of X-ray radiation was a copper anode with a wavelength of 1.5407 Å. To ensure result accuracy and assess potential structure damage or degradation due to radiation, measurements for each sample were replicated, yielding consistent outcomes across all cases.

The initial data went through processing, involving making essential adjustments for background and sample absorption corrections. This results in the determination of the scattered intensity, *I_EXP_*(*Q*), which is expressed as:

$$I_{EXP}{\left(Q\right)}_{E.U}=\overset{N}{\underset{i=1}{\sum }}x_{i}f_{i}^{2}+I\left(Q\right)$$

The independent atomic scattering from the atoms in a stoichiometric unit is represented by the first term. The part of the scattering intensity that is sensitive to the structure, on the other hand, is represented by *I*(*Q*), also referred to as the “total structure function”. This is because it incorporates the contributions of various atoms’ interference.

The transmitted momentum magnitude, denoted by *Q*, is determined by the scattering angle, 2 ϑ, as per the equation *Q* = 4π (sinϑ/λ) [17,18].

The pair correlation functions descriptive of the structure and the function *I*(*Q*) are related in the following formula:

$$I\left(Q\right)=\overset{N}{\underset{i=1}{\sum }}\overset{N}{\underset{j=1}{\sum }}x_{i}x_{j\;}f_{i\;}f_{j}4\pi \rho_{0}\int_{0}^{\infty }r^{2}(g_{ij}\left(r\right)-1)\frac{\sin\left(Q_{r}\right)}{Q_{r}}d$$

(where *f_i_* is the species’ *Q*-dependent X-ray scattering factor, *x_i_* is the species’ numerical concentration, and *ρ*_0_ is the system’s bulk number density).

The modification function $\;M\left(Q\right)=\frac{f^{2}N\left(0\right)}{f^{2}N\left(Q\right)}\exp\left(-0.01Q^{2}\right)$ was multiplied by both the experimental and theoretical structure functions in order to improve the curve resolution at high *Q*. The resulting Fourier transform was then used to create the differential total correlation function *Diff*(*r*), which is based on the relation:

$$Diff\left(r\right)=\frac{2r}{\pi }\int_{0}^{\infty }QI\left(Q\right)\sin\left(rQ\right)M\left(Q\right)dQ$$

In conclusion, comparisons were made between experimental and model data using *QI*(*Q*)*M*(*Q*) and *Diff*(*r*). In examining the attributes of the model, the partial *gij*(*r*) and the total *Diff*(*r*) were taken into account [19,20].

#### 2.2.2. Molecular Dynamic Simulation

The Generalized Amber Force Field (GAFF) was used in simulations to characterize the potential energy [21] with the molecular dynamics engine being the Amber/PMEMD v.12 software [22]. Partial atomic charges, which were determined at the B3LYP/6-31G* level for isolated cations and anions at the equilibrium geometry, were used to describe electrostatic interactions. These charges were obtained by Restrained Electrostatic Potential (RESP) fitting [23]. Using Packmol software volume v20.11.1 [24], initial configurations were produced at random, ensuring a minimum interatomic spacing of 2 Å. Based on the experimental density, the cubic boxes were filled with the required ions and molecules, and their starting edge length was 50 Å—placing 683 ion pairs. The right size of the simulation boxes was guaranteed by selecting an edge length that was twice the average largest correlation distance found in the final observable peak in the experimental Diff(r) distributions (about 25 Å). 

The following succinctly describes the simulation protocol:

- Energy minimizations were achieved through the use of both conjugated gradient and steepest descent methods;
- A brief NVT run (20 ps) at 50 K;
- Gradient heating at 298 K in the NPT ensemble using the Berensen weak coupling algorithm [22], with the external pressure set at 1 atm;
- An approximate 2 ns NPT 298 K equilibration; a productive 2 ns NVT simulation with an integration time step of 2 fs and trajectories collected every 1000 steps;
- Periodic Boundary Conditions were applied to all phases of the calculation in order to minimize the crystal artifacts resulting from finite-size effects.

We used TRAVIS software (version: Feb 23 2016) for hydrogen bond calculations, density distribution function analysis, and pair separation distance analysis. This software allowed us to perform detailed analyses of the molecular interactions and structures within ILs [25].

## 3. Results and Discussion

### 3.1. Experimental and Computational Characterization of Nanostructure

**Figure 2:**
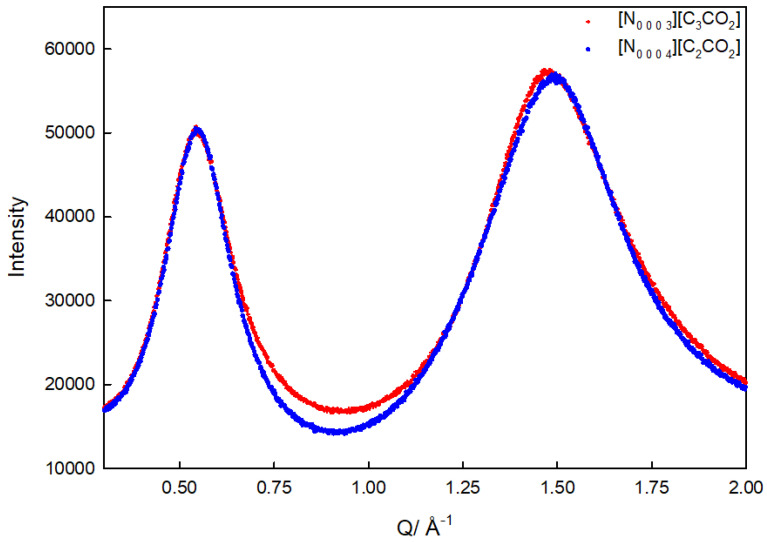
Pre-peak and principal peaks for [N_0 0 0 3_][C_3_CO_2_] (red curve) and [N_0 0 0 4_][C_2_CO_2_] (blue curve).

The investigation of the nanostructural organization of the [N_0 0 0 3_][C_3_CO_2_] and [N_0 0 0 4_][C_2_CO_2_] ionic liquids involved a combination of experimental and computational methodologies. Using small-angle X-ray scattering (SAXS), structural characteristics at various length scales were examined. Within [N_0 0 0 3_][C_3_CO_2_] and [N_0 0 0 4_][C_2_CO_2_], the measured *Q* values, which ranged from 0 to 17 Å^−1^, facilitated the examination of spatial arrangements. The two unique peaks in the scattering pattern, the “principal peak” and the “pre-peak”, offered important new information on how polar and non-polar domains are organized [19]. These peaks, which appeared at particular *Q* values, showed that the liquids possessed well-defined structures, such as the division of polar charged heads from non-polar alkyl chains [26]. The *Q* values of [N_0 0 0 3_][C_3_CO_2_] and [N_0 0 0 4_][C_2_CO_2_] showed an interesting consistency that indicated a constant nanostructure and focus on the reproducibility of these patterns in spite of the variations in alkyl chain lengths (Figure 2).

Molecular dynamics (MD) simulations, an advanced method that models the dynamic behavior of molecules over time, was used in the computational portion of the study. This method gave rise to a complementary understanding of the molecular interactions and arrangements of [N_0 0 0 4_][C_2_CO_2_] and [N_0 0 0 3_][C_3_CO_2_] in both bulk and interfacial circumstances.

Derived from MD simulations, the structure factor QI(Q)M(Q) and the radial distribution function Diff(r) served as essential instruments for the analysis of real and reciprocal spaces, respectively. The distribution of the ions, their interactions, and the dynamic features of their behavior were well explained by these models [27].

A comprehensive knowledge of the nanostructural organization of [N_0 0 0 4_][C_2_CO_2_] and [N_0 0 0 3_][C_3_CO_2_] was achieved through the merging of experimental and computational studies. This is important because ionic liquid behavior at surfaces can diverge significantly from their bulk characteristics [28,29]. Applications in a variety of domains, where interfacial interactions are critical, such as catalysis and energy storage, require an understanding of the nanostructure in both situations.

As a result, the experimental and computational characterization of the nanostructure was a strategic integration rather than a simple comparison of approaches that led to an understanding of the molecular subtleties influencing the behavior of [N_0 0 0 4_][C_2_CO_2_] and [N_0 0 0 3_][C_3_CO_2_]. 

### 3.2. Nanostructural Comparison—Structure Factor and Radial Distribution Function Analysis

This section concentrates on the analysis of the structure factor (QI(Q)M(Q)) and radial distribution function (Diff(r)) derived from SAXS measurements for [N_0 0 0 3_][C_3_CO_2_] and [N_0 0 0 4_][C_2_CO_2_].

The structure of ionic liquids at various length scales was studied using the SAXS method [30,31]. The scattering vector, represented by the measured *Q* values, which vary from 0 to 17 Å^−1^, provides details about the sample’s spatial arrangement [32]. This range enables the investigation of nanostructural characteristics, corresponding to a size domain of approximately 0.4 to 40 nm.

In the SAXS measurements, distinct peaks in the scattering patterns are observed. Two specific peaks are highlighted: the “pre-peak” and the “principal peak”.

**Pre-peak (Q = 0.55 Å^−1^):** This peak is often referred to as the “polarity-polarity peak”. Its occurrence at Q = 0.55 Å^−^¹ in [N_0 0 0 3_][C_3_CO_2_] spectra implies an average size of about 11.42 Å. The interpretation of this peak suggests the separation of non-polar three-carbon alkyl chains of the anion from the polar charged heads. Essentially, this peak indicates the formation of clusters or domains where the polar and non-polar components are segregated, resulting in a nanostructured liquid.

**Principal Peak (Q = 1.47 Å^−1^):** This peak, often referred to as the “adjacency peak,” results from a combination of intermolecular and longer intramolecular distances. In the case of [N_0 0 0 3_][C_3_CO_2_], *Q* = 1.47 Å^−1^, and the correlation distance between the particles is calculated to be 4.3 Å.

A notable observation is that the *Q* values for the positions of the pre-peak and the principal peak for [N_0 0 0 4_][C_2_CO_2_] are identical to those of [N_0 0 0 3_][C_3_CO_2_]. This suggests that, despite differences in alkyl chain lengths, the nanostructure within the system is remarkably similar in both ionic liquids.

Table 1 presents the Q values for the pre-peak and principal peak for both [N_0 0 03_][C_3_CO_2_] and [N_0 0 0 4_][C_2_CO_2_]. The identical values further highlight the consistency in nanostructure between the two ionic liquids.

The consistency in nanostructure emphasizes that, despite variations in alkyl chain lengths, the ionic liquids exhibit similar arrangements of polar and non-polar domains. This result aligns with previous studies on ionic liquids, emphasizing that the nanostructure is primarily influenced by electrostatic forces between charged functional groups and van der Waals force between non-polar alkyl chains [33]. There is a striking similarity between the pre-peak and principal peak positions in [N_0 0 0 4_][C_2_CO_2_] and [N_0 0 0 3_][C_3_CO_2_] in terms of the *Q* values, as indicated in Table 1.

The consistent nanostructure between [N_0 0 0 3_][C_3_CO_2_] and [N_0 0 0 4_][C_2_CO_2_] has significant implications. It suggests that the variation in alkyl chain lengths does not profoundly impact the overall nanostructure of these ionic liquids. This observation aligns with broader studies on ionic liquids with different cation–anion combinations, where the nanostructure is consistently influenced by electrostatic forces [34].

The nanostructural comparison contributes not only to the specific understanding of [N_0 0 0 3_][C_3_CO_2_] and [N_0 0 0 4_][C_2_CO_2_] but also to the broader understanding of ionic liquids. It reinforces the notion that the nanostructure of these liquids is robust and can be maintained despite variations in molecular structure, which is crucial information for tailoring ionic liquids for specific applications. It basically means that the particular configuration and structure at the nanoscale are resilient and stable even when the ionic liquids’ composition varies.

**Figure 3:**
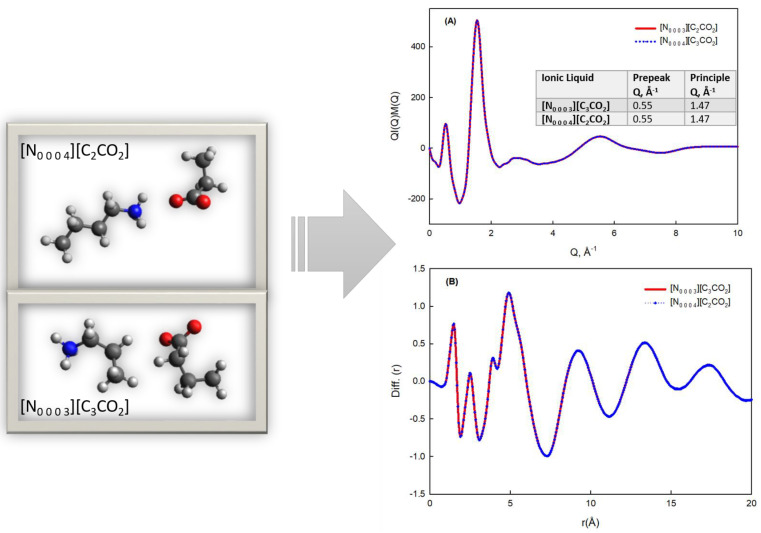
Structure factor (**A**) and radial distribution function (**B**) calculated by MD simulations for [N_0 0 0 3_][C_3_CO_2_] (red curve) and [N_0 0 0 4_][C_2_CO_2_] (blue curve). In (**A**), the inset shows the peak positions.

To further validate the reliability of our MD-simulated structures, we compared the peak positions of the calculated structure factors with those of the experimental data. The alignment of these peaks is a crucial indicator of the accuracy of our simulations. Figure 3 is presented, depicting the structure factor (A) and radial distribution function (B) calculated through MD simulations. The inset in Figure 3A highlights the peak positions of the calculated structure factors, which shows how the peaks in the simulated data correspond to those observed experimentally. MD simulations offer a dynamic perspective on the molecular arrangements and interactions within [N_0 0 0 3_][C_3_CO_2_] and [N_0 0 0 4_][C_2_CO_2_]. The structure factor and radial distribution function derived from MD simulations provide a complementary viewpoint to the experimental results. Confirming the nanostructure observed in experimental data, these simulations offer valuable insights into the dynamic behavior and energetics of the ionic liquids at the molecular level. Through the analysis of structure factors, radial distribution functions, mass density, pair separation distances, and hydrogen bond probabilities, we infer the role of electrostatic and hydrogen bonding interactions in stabilizing the observed nanostructures. While the manuscript does not include direct energy values, the detailed examination of these properties allows us to qualitatively understand the contributions of various interactions to the overall stability and behavior of the system. For instance, the radial distribution function and pair separation distance provide information on the spatial arrangement and preferred distances between ions, which are indicative of the strength and nature of interionic forces. Similarly, the analysis of hydrogen bonds sheds light on the specific interactions contributing to the cohesion and structural integrity of the ionic liquids. By connecting these structural and dynamic properties, we aim to provide a comprehensive understanding of the factors influencing the energetics and behavior of the ionic liquids at the molecular level.

### 3.3. Comparison with Other Alkylammonium Alkanoate Ionic Liquids

The important discovery is that these ionic liquids have a comparable nanostructure even though their alkyl chain lengths varied. This finding is consistent with more general studies conducted on other cation–anion pairings in ionic liquids [35,36] and supports the constant impact of Coulombic forces between charged functional groups on the nanostructural structure of these liquids. This contrast is important, not just for the particular ionic liquids under study but also for the broader knowledge that Coulombic forces are a major factor in forming the nanostructure of ionic liquids.

**Figure 4:**
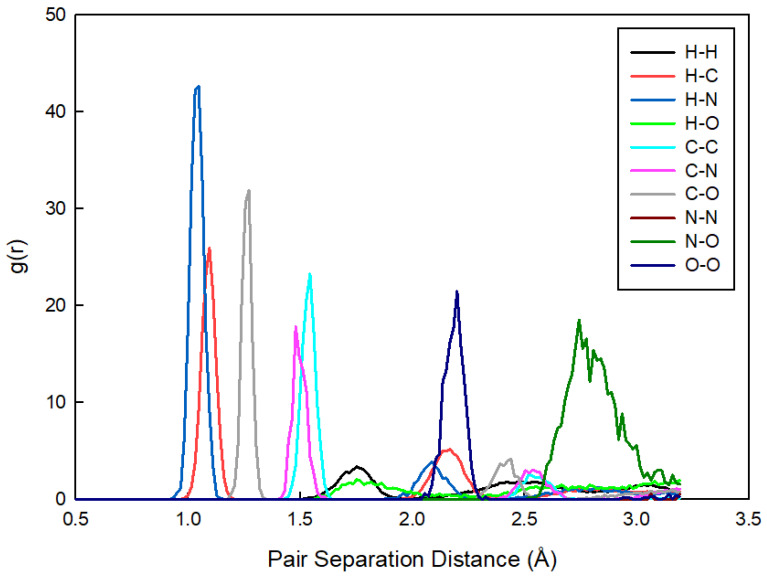
Radial distribution functions g(r) for various pair interactions in the IL [N_0 0 0 3_][C_3_CO_2_], illustrating the probability of finding pairs of particles at different separation distances. This analysis underscores the role of electrostatic forces in determining the nanostructure of the PILs; the values for the [N_0 0 0 4_][C_2_CO_2_] are the same so they are not plotted together.

Figure 4 provides a detailed analysis of the pair separation distances, further illustrating the dominance of Coulombic interactions and the minimal influence of alkyl chain length on the overall nanostructure. The radial distribution functions g(r) for various pair interactions indicate that the electrostatic forces between charged groups primarily determine the nanostructure, which is consistent with our broader findings on the role of Coulombic forces in these systems. The sharp peaks at distances less than 1.5 Å correspond to atoms that are covalently bonded, indicating intramolecular interactions. Broader peaks at distances greater than 1.5 Å indicate weaker, non-covalent intermolecular (interionic) interactions, such as electrostatic forces between ions.

### 3.4. Density Analysis

The comparison of theoretical densities was obtained through molecular dynamics simulations with experimental densities [37]. Table 2 presents the experimental and simulated densities for [N_0 0 0 3_][C_3_CO_2_] and [N_0 0 0 4_][C_2_CO_2_]. The slight variations between experimental and simulated densities prompt an exploration of factors influencing these differences. While the parametrization of the force field is the most probable cause, as it directly impacts the accuracy of molecular dynamics simulations, other factors such as temperature, pressure, water content, and packing fraction (the ratio of the volume occupied by ions in the system to the total volume of that system) can also affect the effective hard-core diameter of ions and should be considered [38,39]. The analysis acknowledges that certain factors not captured by theoretical models, such as system size effects, voids, and pores in the composites, contribute to the observed differences [40]. 

This consideration enhances the understanding of the complexities involved in accurately predicting the density of ionic liquids, highlighting the need for a comprehensive analysis that accounts for various influencing factors.

Simulated and experimentally determined values are not in exact agreement, but the theoretical data are ca. 1–3% higher. The “theoretical” densities of the present calculations (i.e., the simulation box densities obtained at the end of the NPT phase) have this result, but there was reasonable agreement because the cell volume was not adjusted in the production phase.

**Figure 5:**
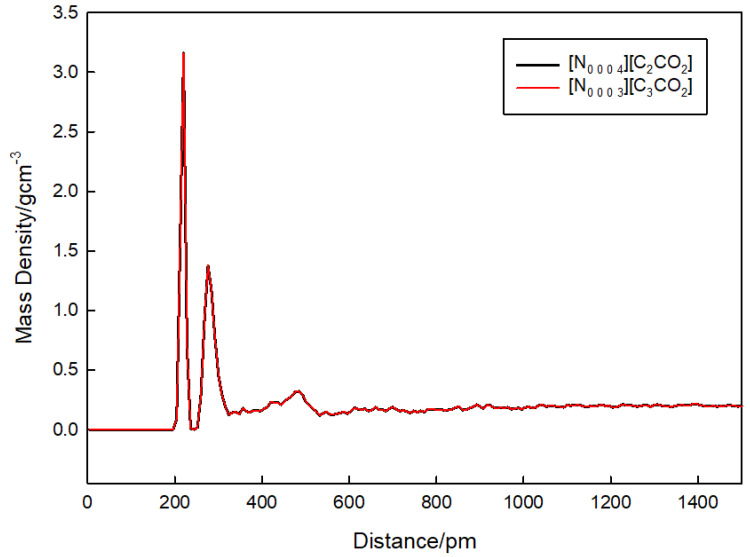
Mass density distribution as a function of distance for [N_0 0 0 3_][C_3_CO_2_] (red line) and [N_0 0 0 4_][C_2_CO_2_] (black line).

Figure 5 further supports these findings by illustrating the mass density distribution as a function of distance for both [N_0 0 0 3_][C_3_CO_2_] and [N_0 0 0 4_][C_2_CO_2_]. The figure shows sharp peaks at short distances, indicating regions of high mass density close to the ionic core. These sharp peaks at distances around 200 pm suggest strong local ordering of the ions within the PILs. Additionally, there are secondary peaks around 400 pm, which likely correspond to the first solvation shell around the central ion. The density profiles for both [N_0 0 0 3_][C_3_CO_2_] (red line) and [N_0 0 0 4_][C_2_CO_2_] (black line) are very similar, which supports the finding that the nanostructure of these PILs is primarily determined by electrostatic interactions and is not significantly influenced by the length of the alkyl chain. The similarity in the density profiles indicates that the nanostructure is primarily determined by Coulombic forces, with minimal influence from the variation in alkyl chain length.

### 3.5. Effect of Dilution on the Nanostructure

Properties and behavior of ionic liquids can be influenced by the presence of water, which is often unavoidable in practical situations. Therefore, it is important to understand how water affects the nanostructure of ionic liquids, which is the arrangement of ions and molecules at the nanometer scale.

The impact of water on the nanostructure of [N_0 0 0 3_][C_3_CO_2_] and [N_0 0 0 4_][C_2_CO_2_] was investigated, and the results highlight the consistent effects observed within the alkylammonium alkanoate class of ionic liquids [27]. When water is introduced into these ionic liquids (ILs), the influence varies depending greatly on the concentration. At low water concentrations, the water molecules tend to exist independently within the ILs. However, as the concentration of water increases, a shift occurs, leading to the formation of a network among water molecules. This self-aggregation of water molecules at different molar fractions correlates with the organization of the ionic liquid into a polar network.

**Figure 6:**
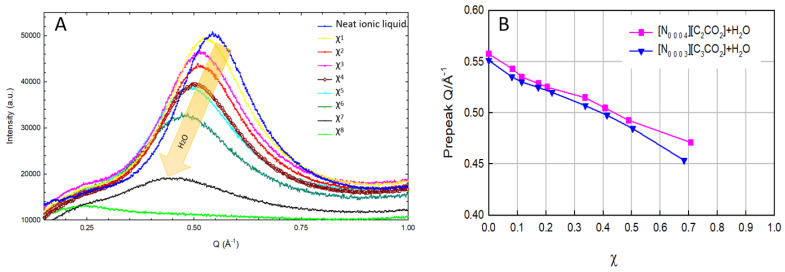
Pre-peak positions upon addition of water on [N_0 0 0 3_][C_3_CO_2_] and [N_0 0 0 4_][C_2_CO_2_] (**A**) and peak positions with respect to their water mixtures (**B**).

The introduction of water to [N_0 0 0 3_][C_3_CO_2_] and [N_0 0 0 4_][C_2_CO_2_] is observed to disrupt the interactions between the cations and anions within the liquid. This disruption leads to a weakening of Coulombic interactions between charged species. Figure 6 presents pre-peaks, which are considered indicative of the presence of nanostructure in the ionic liquids. In Figure 6A, different concentrations of water in the ionic liquids are depicted, and the arrow in the spectra indicates an increasing concentration from top to bottom. The mole fraction of water, χwater, is used to determine the compositions of the ionic liquid–water mixes. The variations in χwater are represented by χ^0^ to χ^8^, where each corresponds to an ionic liquid–water mixture with a specified χwater. Additionally, χ^0^ corresponds to the neat ionic liquid. Legends have been added to the figure, providing a clear indication of χ^0^ (no water, or neat ionic liquid) to χ^8^. Observations from the figure reveal that as the concentration of water increases, the pre-peaks start disappearing as shown in Figure 6B. In the spectrum of the pure ionic liquid (at the top peak), the absence of any observable peak or aggregation indicates that it is simply water without any ionic liquid present (at the bottom peak).

This phenomenon implies that the ionic liquid creates distinct regions characterized by varying degrees of polarity. This variation is contingent on how the charged and non-charged components of the ions are distributed within the system. The interplay between water and the ionic liquid is explored through various techniques, including molecular dynamics simulations [15], Raman [41] and FTIR spectroscopy [42], vibrational spectroscopy [43], as well as small-angle and wide-angle X-ray scattering techniques [30]. These techniques collectively provide insights into the structure, dynamics, and interactions occurring between the ionic liquid and water molecules across different length and time scales. The investigation contributes to a comprehensive understanding of how the introduction of water influences the nanostructure of ionic liquids, shedding light on their intricate behavior and organization.

**Figure 7:**
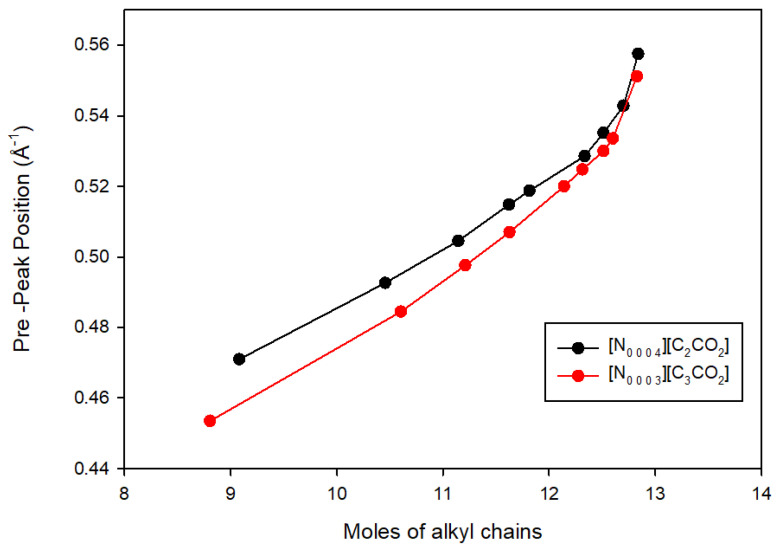
Alkyl chain concentrations vs. pre-peak positions in [N_0 0 0 3_][C_3_CO_2_] and [N_0 0 0 4_][C_2_CO_2_].

In Figure 7, the relationship between the concentrations of alkyl chains and pre-peak positions in [N_0 0 0 3_][C_3_CO_2_] and [N_0 0 0 4_][C_2_CO_2_] is depicted, offering a distinctive perspective on the nanostructural patterns. Unlike Figure 6, which focused on water concentration, this figure illustrates how an increase in alkyl chain concentrations influences pre-peak positions. The x-axis represents moles of increased alkyl chains, which is crucial for indicating the concentration of these structures within the ionic liquids. The y-axis reflects pre-peak positions, signifying heterogeneities in the nanostructure. The key concept conveyed is that heightened alkyl chain concentrations correlate with shifts in pre-peak positions, indicating variations in nanostructural patterns. This aligns with the overarching theme observed in Figure 6, where the introduction of water impurities led to changes in peak positions, collectively underscoring the impact of impurities in altering the nanostructural organization of these protic ionic liquids.

While the focus is on the impact of water, the statement also highlights the broader perspective of studying mixtures of ionic liquids with other solvents. Exploring these mixtures is seen as essential to broaden the industrial and technological applications of ionic liquids.

**Figure 8:**
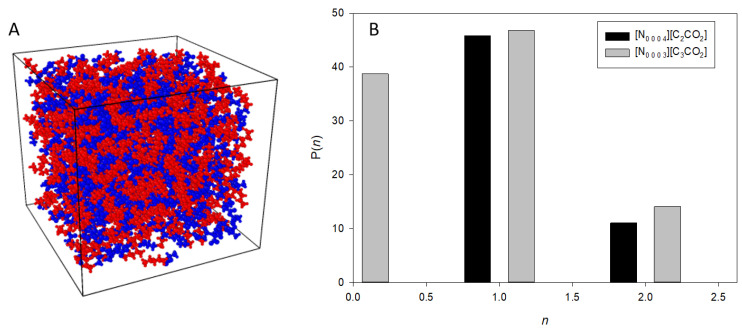
(**A**): Simulation box of [N_0 0 0 3_][C_3_CO_2_] showing the spatial distribution of ions, with red representing anions and blue representing cations. (**B**): Probability distribution P(n) of hydrogen bonds formed in the most dilute solutions of the protic ionic liquids [N_0 0 0 4_][C_2_CO_2_] (black bars) and [N_0 0 0 3_][C_3_CO_2_] (gray bars). The *x*-axis represents the number of hydrogen bonds *n*, and the *y*-axis represents the probability P(*n*) of observing each hydrogen bonding configuration. Both ionic liquids predominantly form one hydrogen bond (*n* = 1), with notable probabilities for zero (*n* = 0) and two (*n* = 2) hydrogen bonds.

To understand the hydrogen bonding behavior in the most dilute solutions of these ionic liquids, we analyzed the probability distribution P(n) of hydrogen bonds formed. Figure 8 illustrates this distribution for [N_0 0 0 4_][C_2_CO_2_] and [N_0 0 0 3_][C_3_CO_2_]. The dominant configuration involves the formation of a single hydrogen bond (*n* = 1), while significant populations also exhibit no hydrogen bonds (*n* = 0). Occasionally, molecules form two hydrogen bonds (*n* = 2). The similarities in the distributions for [N_0 0 0 4_][C_2_CO_2_] and [N_0 0 0 3_][C_3_CO_2_] further highlight the consistent hydrogen bonding characteristics, irrespective of the specific ionic liquid structure. Both ionic liquids predominantly form one hydrogen bond (*n* = 1), with notable probabilities for zero (*n* = 0) and two (*n* = 2) hydrogen bonds. The overall distribution shapes for [N_0 0 0 4_][C_2_CO_2_] and [N_0 0 0 3_][C_3_CO_2_] are similar, with [N_0 0 0 4_][C_2_CO_2_] having slightly higher probabilities for *n* = 0 and *n* = 2, while [N_0 0 0 3_][C_3_CO_2_] shows a higher probability at *n* = 1. This indicates that in a highly dilute solution, many molecules are isolated or weakly interacting, and there is occasional formation of multiple hydrogen bonds. These insights into the hydrogen bonding probabilities showcase the prevalence of single hydrogen bond formations in highly dilute solutions and emphasize the robustness of hydrogen bonding behavior across different PILs.

In our analysis using TRAVIS, we specified the hydrogen bond parameters with the donor molecule being [N_0 0 0 4_]^+^ and the acceptor molecule being [C_2_CO_2_]^−^. Specifically, the nitrogen (N) atom in [N_0 0 0 4_]^+^ acted as the hydrogen bond donor, while the oxygen (O) atom in [C_2_CO_2_]^−^ served as the hydrogen bond acceptor. The threshold values for detecting hydrogen bonds were set to a maximum distance of 245 pm (2.45 Å) between the hydrogen atom and the accepting atom, 350 pm (3.50 Å) between the donating atom and the accepting atom, and a maximum angle of 30° between the donor–acceptor and donor–hydrogen atoms. These criteria ensured precise identification of hydrogen bonds within our system.

## 4. Conclusions

This paper investigates the nanostructure of two PILs, [N_0 0 0 3_][C_3_CO_2_] and [N_0 0 0 4_][C_2_CO_2_], which have similar polar head groups (NH_3_^+^ and COO^−^), but different alkyl chain lengths and positions on the head groups. The approach proposed here uses two methods to study the nanostructure of these PILs: the X-ray scattering technique and molecular dynamics simulations. The bulk properties of the PILs, e.g., density, are affected by the presence of nanostructural organizations in these systems. The nanostructure of the PILs was found to be mainly determined by the electrostatic forces between the charged functional groups ([N*R*H_3_]^+^ and [C*R*_2_OO]^−^), which form strong ionic bonds and create ordered regions in the ILs. The polar head groups play a dominant role in shaping the nanostructure of the PILs, while the alkyl chain lengths and positions have little effect on the nanostructure. This paper shows that despite having different alkyl chain lengths in both cation and anion, the PILs exhibit remarkably similar nanostructures in both bulk and interfacial phases. Thus, this research provides a combined experimental and theoretical comprehensive understanding of the nanostructure of two ionic twin PILs.

The introduction of water to the [N_0 0 0 3_][C_3_CO_2_] and [N_0 0 0 4_][C_2_CO_2_] disrupts the interactions between the cations and anions within the liquid, leading to a weakening of Coulombic interactions between charged species. As the concentration of water increases, the nanostructure indicated by the pre-peaks in the spectra diminishes, reflecting the disruption of the ordered regions in the PILs. This trend shows that higher water content leads to a loss of the characteristic nanostructure of the neat ionic liquids.

## Figures and Tables

**Table 1:** Pre-peak and principal Q values.

Ionic Liquid	Pre-Peak (Q1), Å^−1^	Correlation Distance (d1), Å	Principal Peak (Q2), Å^−1^	Correlation Distance (d2), Å
[N_0 0 0 3_][C_3_CO_2_]	0.55	11.42	1.47	4.27
[N_0 0 0 4_][C_2_CO_2_]	0.55	11.42	1.47	4.27

**Table 2:** Experimental and theoretical densities.

Ionic Liquid	Densities Density (g/cm^3^) Experimental Simulated		Percentage Difference
[N_0 0 0 3_][C_3_CO_2_]	0.9441	0.9599	1.66%
[N_0 0 0 4_][C_2_CO_2_]	0.9450	0.9556	1.12%

## Data Availability

The raw data supporting the conclusions of this article will be made available by the authors on request.
